# Development of categorical speech perception in Mandarin‐speaking children and adolescents

**DOI:** 10.1111/cdev.13837

**Published:** 2022-08-03

**Authors:** Yan Feng, Gang Peng

**Affiliations:** ^1^ School of Foreign Studies Nanjing University of Science and Technology Nanjing China; ^2^ Department of Chinese and Bilingual Studies, Research Centre for Language, Cognition, and Neuroscience The Hong Kong Polytechnic University Hong Kong SAR China; ^3^ Shenzhen Institutes of Advanced Technology Chinese Academy of Sciences Shenzhen China

## Abstract

Although children develop categorical speech perception at a very young age, the maturation process remains unclear. A cross‐sectional study in Mandarin‐speaking 4‐, 6‐, and 10‐year‐old children, 14‐year‐old adolescents, and adults (*n* = 104, 56 males, all Asians from mainland China) was conducted to investigate the development of categorical perception of four Mandarin phonemic contrasts: lexical tone contrast Tone 1‐2, vowel contrast /u/−/i/, consonant aspiration contrast /p/−/p^h^/, and consonant formant transition contrast /p/−/t/. The results indicated that different types of phonemic contrasts, and even the identification and discrimination of the same phonemic contrast, matured asynchronously. The observation that tone and vowel perception are achieved earlier than consonant perception supports the phonological saliency hypothesis.

**Abbreviations**
CP: categorical perception
F0: fundamental frequency
F1: the first formant
F2: the second formant
LMM: linear mixed‐effect model
MMN: mismatch negativity
VOT: voice onset time

## INTRODUCTION

The brain weight and synaptic density of newborn infants grow dramatically in the first 2 years of life (Purves et al., 2013), and these age‐related developments provide a physiological basis for language acquisition in a universal timeline (Kuhl & Damasio, 2012). According to that timeline, infants have learned to perceive language‐specific phonemes, to understand 60–90 words, and to produce the first word in their native language by the end of their first year. During their second year, they have learned to comprehend basic language‐specific word order and to produce 200–300 words. By the end of their third year, they can understand some complex sentences and can produce 1000 words, different tenses, and adult‐level sentence construction (Kuhl & Damasio, 2012).

In daily communication, speech consists of continuous acoustic cues that are highly variable. Speech perception is an essential skill that children need to acquire to communicate with others (Denes & Pinson, 2015). One aspect of speech perception is the need for individuals to decode acoustic cues into discrete phonemes via so‐called categorical perception (CP; Liberman et al., 1957). It has been reported that the CP abilities of 6‐month‐old infants can predict their language development during their first 2 years (Tsao et al., 2004). Thus, acquiring CP is an important and basic stage of language development for children.

In the classical paradigm of CP (Liberman et al., 1957), there are two types of tasks—identification and discrimination—which reflect different phonological processing abilities and require different cognitive resources (Fujisaki & Kawashima, 1971; Xu et al., 2006). For phonemic identification, listeners need to decode one sound stimulus and map the sound to long‐term developed phonemic knowledge/categories. Identification requires sensory memory and long‐term phonological memory (Xu et al., 2006). For phonemic discrimination (e.g., the AX pattern), listeners need to memorize the first sound until the release of the second sound and then compare the two sounds. Thus, phonemic discrimination places a higher demand on working memory than identification does, and the process of phonemic discrimination is more complex and requires greater cognitive resources (Feng et al., 2021). Both within‐category and between‐category discrimination require sensory memory and working memory, and between‐category discrimination additionally recruits long‐term phonological memory (Xu et al., 2006).

Repp (1984) described several CP characteristics with respect to identification and discrimination performance. The identification curve should have a steep change from one phonological category to another. In the discrimination curve, a peak of discrimination accuracy should appear at the position of the category boundary. In addition, between‐category accuracy should be significantly higher than within‐category accuracy is, and within‐category accuracy should be close to the chance level (50%). Eimas et al. (1971) reported that infants aged 1 and 4 months showed evidence of CP capability for English voiced and voiceless stops (/b/−/p/).

Infants after birth can discriminate a wide range of native and non‐native speech contrasts. With exposure to their native language, their sensitivity to native speech contrasts increases and that to non‐native ones decreases (Kuhl et al., 2008). Accumulated evidence has also shown that such perceptual reorganization happens over the first year, and thus infants can perceive language‐specific phonemic contrasts at a very early age. Kuhl et al. (1992) found that English‐ and Swedish‐learning infants at 6 months of age showed asymmetric discrimination of the English vowel /i/ and the Swedish vowel /y/. Native language experience by 6 months of age can lead to a language‐specific categorization of vowels. Polka and Werker (1994) also observed that English‐learning infants at 4 months of age could discriminate both the native English vowel contrast /i/−/a/ and non‐native German vowel contrasts /U/−/Y/ and /u/−/y/, but the non‐native German vowel discrimination declined in infants at 6–8 months of age. That study also reported that the shift from language‐general to language‐specific perception began earlier for vowels than for consonants. Werker and Tees (1984) found that English‐learning infants younger than 10 months of age could discriminate non‐native Salish and Hindi consonant contrasts, but they lost that ability at 10–12 months of age. Best et al. (1988) further clarified that such language‐specific perceptual reorganization in infants was a function of phonological development that assimilated speech sounds to phonemic categories in the mother tongue. However, for non‐native sounds (e.g., Zulu click consonants) that are impossible to be assimilated to native categories, Best et al. (1988) reported that English‐speaking adults and infants showed a similar ability to discriminate the sounds purely on the basis of auditory properties.

In addition to vowels and consonants, the acquisition of prosody (e.g., lexical tone and stress) has also received attention. Singh et al. (2018) demonstrated that Mandarin monolinguals could discriminate the salient tone contrast Tone 1‐Tone 3 at 6 months, and they could discriminate the subtle tone contrast Tone 2‐Tone 3 at 9 months. Moreover, the acquisition of lexical tones differs in different language environments. Infants aged 6 and 9 months old from a tonal (Chinese) language environment have been reported to perform equally well in lexical tone discrimination, whereas the ability of lexical tone discrimination in infants from a non‐tonal (English) language environment declined at 9 months of age relative to 6 months of age (Mattock & Burnham, 2006).

The acquisition of stress patterns is also language specific (Höhle et al., 2009; Skoruppa et al., 2009). Skoruppa et al. (2009) found that 9‐month‐old Spanish infants could perceive stress patterns at the phonological level, whereas French infants discriminated stress patterns at the acoustic level. Jusczyk et al. (1993) observed that English‐learning infants acquired a preference for a predominant stress pattern between the ages of 6 and 9 months. Höhle et al. (2009) also reported that German infants acquired such preference between the ages of 4 and 6 months, whereas French infants showed no such bias.

Put together, Kuhl (2004) proposed a universal language timeline for the development of speech perception in infants, along which the infants develop language‐specific perceptual ability at 6 months. More specifically in regard to the perception of segments, vowel perception develops earlier (at approximately 6 months old) than consonant perception does (at approximately 11 months old). The perception of suprasegments, such as stress patterns, develops at roughly 8 months of age (Kuhl, 2004).

Nonetheless, the point at which infants or children obtain a mature, adult‐level ability to categorically perceive segments and suprasegments remains unclear. It is important to distinguish phonological emergence, phonological stabilization, and phonological maturation in progressive language development. According to Peng and Chen (2020), the first time that infants can perceive and produce a phonetic unit is referred to as the age of emergence. Stabilization refers to the child's ability to perceive and produce speech sounds with relatively high accuracy. Maturation reflects children being able to perceive and produce speech sounds similarly to adults.

Another crucial issue is the developmental order of CP for different types of phonemic contrasts. Jakobson (1968) proposed that language acquisition in children occurs in an ordered way, and Kuhl's (2004) timeline also indicates that the perceptual development of different types of phonemic contrasts is asynchronous. However, differences in the populations and language backgrounds investigated in previous studies hinder a direct comparison across multiple phonemic contrasts. Therefore, it should be helpful to examine the developmental order of multiple phonemic contrasts within a single study with the same group of participants. Indeed, there has been limited research on CP that has directly compared multiple phonemic contrasts in children. Therefore, this study aimed to uncover the maturation of CP in Mandarin‐speaking children and adolescents, and to explore the developmental order of CP for different types of phonemic contrasts.

Table 1 lists some findings on the maturation of CP in children and adolescents. Previous cross‐language research has reported a universally long and slow maturation process for the perception of segments. Ohde and German (2011) assessed the development of vowel perception (/bi/−/bɑ/) in English‐speaking children between the ages of 3 and 4 years and found that the children showed a different vowel perceptual process with a differing category boundary and shallower slope, compared with adults. Arai et al. (2008) investigated the identification of Japanese vowel duration (/a:/−/a/) in children aged 5 to 8 years and found a significant developmental improvement from 5‐year‐old children to those aged six, and the identification performance of 7‐year‐old children was similar to that of adults. For consonant perception, Walley and Carrel (1983) investigated the identification of English consonants (/ba/−/da/−/ga/; /bu/−/du/−gu/) in native 4‐ to 6‐year‐old children and adults and found that the children showed an overall performance that was identical to that of the adults. However, Sussman (1993) also compared the perceptual development of consonant formant transition relative to the place of articulation (/ba/−/da/) in English‐speaking children between the ages of four and six and found that neither their discrimination nor identification abilities were mature. Flege and Eefting (1986) examined the perception of English voiceless and voiced stops (/t/−/d/) in 10 children at the age of 9 years, whose mother tongue was English or Spanish. They found that age and language background affected the perception of stops, and in those languages, 9‐year‐old children had not obtained an adult‐level perceptual ability. Hazan and Barrett (2000) examined the perceptual development of four phonemic contrasts (i.e., /g/−/k/, /d/−/g/, /s/−/z/, and /s/−/ʃ/) in English‐speaking children between the ages of 6 and 12 years and observed a significant increase in the steepness of the identification gradient from the children aged six to those aged 12 years. However, the children at the age of 12 still did not have a mature perception of these phonemic contrasts, in comparison with adults, and they also lacked perceptual flexibility when they encountered phonemes with signal degradation. Medina et al. (2010) compared the development of voicing perception (/də/−/tə/) in French‐speaking children with a mean age of 9 years and adolescents aged 17 years. The children showed a shallower slope with non‐perfect identification scores at the two extremes in the identification function, whereas the adolescents could identify the voicing contrast as well as adults, thus indicating that the participants' identification ability matured during the period from nine to 17 years of age.

**TABLE 1:** Studies on the development of categorical speech perception (all studies except Lee et al., 2012 involved an adult control group)

Studies	Sample size	Age range	Mother tongue	Phonemes	Age of maturation
Walley and Carrel (1983)	46	4‐ to 6‐year‐old children	English	Consonants (/ba/−/da/−/ga/; /bu/−/du/−/gu/)	Identification: 4–6 years old
Flege and Eefting (1986)	40	9‐year‐old children	English; Spanish	Consonants (/ta/−/da/)	Identification: not yet
Sussman (1993)	30	4‐ to 6‐year‐old children	English	Consonants (/ba/−/da/)	Identification: not yet Discrimination: not yet
Hazan and Barrett (2000)	97	6‐ to 12‐year‐old children	English	Consonants (/g/−/k/, /d/−/g/, /s/−/z/, /s/−/ʃ/)	Identification: not yet
Arai et al. (2008)	150	5‐ to 8‐year‐old children	Japanese	Vowels (/a/−/a:/)	Identification: 7 years old
Xi et al. (2009)	71	5‐ to 7‐year‐old children	Mandarin	Lexical tones (Tone 1‐2)	Identification: 6 years old
				Consonants (/pa/−/p^h^a/)	Identification: not yet
Medina et al. (2010)	69	8‐ to 11‐year‐old children and 17‐year‐old adolescents	French	Consonants (/də/−/tə/)	Identification: 17 years old Discrimination:8–11 years old
Ohde and German (2011)	20	3;8‐ to 4;1‐year‐old children	English	Vowels (/bi/−/bɑ/)	Identification: not yet
Lee et al. (2012)	50	4‐ to 6‐year‐old children	Taiwanese Mandarin	Lexical tones (Tone 1‐2‐3)	Discrimination: not yet
				Vowels (di‐da‐du)	Discrimination: 6 years old
				Consonants (ba‐da‐ga)	Discrimination: not yet
Liu et al. (2014)	64	3‐ to 8‐year‐old children	Mandarin	Lexical tones (Tone 2‐3)	Discrimination: not yet
				Consonants (/tɕ^h^/−/ɕ/)	Discrimination: not yet
Chen et al. (2017)	86	4‐ to 7‐year‐old children	Mandarin	Lexical tones (Tone 1‐2)	Identification: 6 years old Discrimination: not yet
Ma et al. (2021)	61	4‐ to 6‐year‐old children	Mandarin	Lexical tones (Tone 1‐2)	Identification: 6 years old Discrimination: 6 years old
				Consonants (/pa/−/p^h^a/)	Identification: not yet Discrimination: not yet

It is important to note that the studies mentioned above focused primarily on the perception of segments in children whose native languages were non‐tonal languages. In contrast, limited attempts have been made to examine the development of CP in Mandarin‐speaking children. Mandarin is a tonal language with four lexical tones that differ in pitch patterns; tone as a suprasegment can distinguish different lexical meanings. For example, the syllable /mi/ means: “眯squint” with the high‐level tone (Tone 1), “迷confuse” with the mid‐rising tone (Tone 2), “米rice” with the falling‐rising tone (Tone 3), and “秘secret” with the falling tone (Tone 4; Chao, 1968). Accumulated evidence has shown that tone perception in listeners with tonal language experience differs from that in listeners with non‐tonal language experience (e.g., Mattock & Burnham, 2006; Peng et al., 2010; Xu et al., 2006). Chen et al. (2017) compared the CP of Mandarin lexical tones (Tone 1‐Tone 2) in children aged 4 to 7 years and observed that 6‐year‐old children could clearly identify tones in the same categorical way that adults could. However, although the significant correlation between age and discrimination accuracy revealed that older children showed a trend for better between‐category discrimination, the children at the age of seven had not acquired an adult‐level ability to discriminate tones. The different developmental courses of identification and discrimination abilities reported in Chen et al. (2017) suggest that it is necessary to assess children's identification and discrimination abilities simultaneously.

Another special feature of Mandarin is consonant aspiration, which is distinct from the voiced and voiceless contrast in English. There are six pairs of unaspirated and aspirated consonants that differ in voice onset time (VOT). Some studies have attempted to identify the developmental order of CP for Mandarin segments and suprasegments. Xi et al. (2009) investigated the identification of Mandarin tones (Tone 1‐Tone 2) and consonant aspiration (/pa/−/p^h^a/) in children aged 5 to 7 years and found that the children at the age of six had acquired an adult‐level CP of Mandarin tones, whereas the CP of consonant aspiration in the 7‐year‐olds was not mature. Those findings revealed that the CP of Mandarin tone and consonant aspiration follows different developmental courses, but that study did not explore the development of phonemic discrimination. Recently, Ma et al. (2021) compared the development of identification and discrimination of Mandarin tones and consonant aspiration in children aged 4 to 6 years and found that the children reached an adult level of CP for lexical tones at the age of six. However, the 6‐year‐old children could not identify or discriminate the consonant aspiration in the categorical way that adults could. Thus, tone perception development appears to occur earlier than aspiration perception does.

Whereas the research discussed above was based on traditional behavioral experiments, a few studies have investigated the CP for Mandarin‐speaking children physiologically. Lee et al. (2012) recorded pre‐attentive cortical mismatch responses to tones (Tone 1‐Tone 2‐Tone 3), vowels (di‐da‐du), and initial consonants (ba‐da‐ga) in Taiwanese Mandarin‐speaking children aged 4–6 years. They found that the 6‐year‐old children showed mature mismatch negativity (MMN) patterns, which were elicited by small deviant vowel stimuli. Adult‐level MMN was elicited only by large deviant tone stimuli in all children, and no MMN was found in initial consonant perception. Their findings support the observation that the maturation of phoneme discrimination is a long and slow process in preschool‐aged children.

Lee et al.'s (2012) results also support the phonological saliency hypothesis (Zhu & Dodd, 2000) that phonemes with higher saliency are acquired earlier in children. Three rules determine the saliency of phonemes: (1) in a specific language, a compulsory phoneme is more salient than an optional phoneme (e.g., in Mandarin, a syllable without a vowel [compulsory] would be illegal, but a syllable without a consonant [optional] could be legal), (2) a phoneme that can distinguish different meanings of words is more salient than other phonemes, and (3) a phoneme with fewer permissible choices is more salient than others (e.g., there are fewer tones than consonants in Mandarin, meaning that tone has fewer permissible choices). In Mandarin, all tones, vowels, and consonants can distinguish lexical meanings. Both Mandarin tones and vowels are compulsory in syllabic structure, whereas consonants are optional. In addition, there are 21 permissible initial consonants, which is more than the number of permissible tones and vowels (five vowel phonemes in Li & To, 2017, or nine vowel phonemes in Zhu & Dodd, 2000). Therefore, the saliency of Mandarin tones and vowels is greater than that of initial consonants. Zhu and Dodd (2000) proposed that the lower saliency of consonants in the Mandarin phonological system is responsible for the later acquisition of consonants in children.

### The present study

This study was primarily exploratory. Although CP development has been investigated empirically, many studies have failed to identify the age of maturation for different contrasts, as is shown in Table 1. The different populations and language backgrounds studied in previous research also have hindered a direct comparison. Therefore, this is the first study to systematically investigate the maturation of CP for Mandarin segments and suprasegments in the same sample of children and adolescents. We aimed to assess identification and discrimination abilities for four types of phonemic contrasts in Mandarin‐speaking 4‐, 6‐, and 10‐year‐old children and 14‐year‐old adolescents. The behavioral performance of the children and adolescents (the target groups) was compared directly with that of young adults (the control group). If CP in the target groups was immature, there would be a significant difference between the target groups and the control group. Otherwise, if the target groups had acquired a mature CP ability that was comparable with that of the control group, we would not observe a significant difference between the target and control groups.

We explored four types of Mandarin phonemic contrasts with different temporal or spectral and static or dynamic acoustic features (see more details in Table S1). Tone 1 and Tone 2 are suprasegments that differ primarily in fundamental frequency (F0), a spectral acoustic cue which has a relatively low frequency and changes slowly. Monophthongs /u/ and /i/ are segments that differ mainly in formants, which are spectral and static acoustic cues that have a relatively high frequency and change slowly. Two consonant contrasts were examined in this study—consonant aspiration contrast /p/−/p^h^/ and consonant formant transition contrast /p/−/t/. On one hand, consonant aspiration is a special feature in Mandarin and has been reported to be challenging to children and non‐native learners (Ma et al., 2021; Xi et al., 2009). Consonant aspiration with differing VOTs is a temporal and dynamic acoustic cue with a rapid change. On the other hand, the consonant formant transition associated with the articulatory place of the preceding consonant is a spectral and dynamic acoustic cue with a high frequency that changes rapidly. Therefore, we assessed the perceptual development of the two consonant contrasts simultaneously.

## METHOD

### Participants

A total of 104 participants were recruited in this study: twenty 4‐year‐old children (11 males) from kindergarten in October 2019, twenty 6‐year‐old children (10 males) and twenty 10‐year‐old children (10 males) from primary school in November 2017, twenty 14‐year‐old adolescents (10 males) from middle school in April 2018, and 24 young adults (15 males, 20–30 years old) in September 2017. The young adults and the parents of the children and adolescents were from northern China. All the participants were native Mandarin speakers, none of whom reported any hearing, language, or cognitive impairment. Three children aged 4 years and one child aged 6 years failed to complete the tasks because of fatigue and were excluded from further data analysis. Consent forms with the study protocol approved by the Human Subjects Ethics Subcommittee of The Hong Kong Polytechnic University were obtained from all the young adults and the parents of the children and adolescents. The selection of ages was built upon previous studies, as shown in Table 1. First, most of the previous studies had chosen the ages of 4 and 6 years (e.g., Chen et al., 2017; Lee et al., 2012; Ma et al., 2021; Sussman, 1993; Walley & Carrel, 1983), and some of the studies had found that their identification ability for some vowels and lexical tones was mature at the age of six, so we also selected the ages of four and six. Second, some of the studies had observed that the identification and discrimination of consonants were not mature in children between the ages of 7 and 9 years (e.g., Chen et al., 2017; Flege & Eefting, 1986; Liu et al., 2014; Xi et al., 2009), so we chose the age of 10. Third, Hazan and Barrett (2000) had found that the identification of consonants in 12‐year‐old children still was not mature, so we selected the age of 14. Moreover, because the age gap in Medina et al. (2010) was relatively large (6 years), having examined 8‐ to 11‐year‐old children and 17‐year‐old adolescents, the selection of the age of 14 in the current study could also help narrow the age gap in Medina et al. (2010).

### Materials

We selected four pairs of contrasting words (“衣” /i/ with Tone 1, *clothes*—“姨” /i/ with Tone 2, *aunt*; “嘟” /tu/, *toot*—“滴” /ti/, *drip*; “八” /pa/, *eight*—“趴” /p^h^a/, *grovel*; and “八” /pa/, *eight*—“搭” /ta/, *build*) that are frequently used in Chinese. The level tone is a static acoustic cue, whereas the contour tone is a dynamic cue. Therefore, we assessed the Tone 1 (level tone)‐Tone 2 (contour tone) contrast in this study. The F0 differences of tones happen in a low frequency. To investigate the age‐related differences in both low‐frequency and high‐frequency processing, we then chose /u/ and /i/, which differ mainly in the second formant, and both /u/ and /i/ are high vowels differing in vowel backness: /i/ is a front vowel and /u/ is a back vowel. Also, Lee et al. (2012) had compared the contrasts of /a/−/i/ and /a/−/u/ without the contrast of /u/−/i/, so we chose the contrast between /u/ and /i/ to fill the gap. For consonants, previous studies had reported that children could acquire the production of /p/, /p^h^/, and /t/ before the age of 4 years (e.g., Zhu & Dodd, 2000), so for this study we chose the contrasts between /p/ and /p^h^/ for consonant aspiration, and the contrasts between /p/ and /t/ for consonant formant transition, which might be more feasible for young children. Furthermore, these contrasts had also been used in some previous studies, as listed in Table 1, making it potentially helpful to compare our findings with previous ones.

**FIGURE 1:**
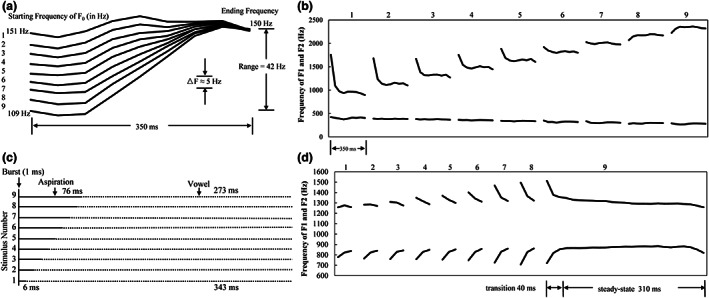
Schematic diagrams of (a) F0 contours in the tone continuum, (b) formants in the vowel continuum, (c) voice onset time in the aspiration continuum, and (d) consonant formant transitions in the transition continuum.

The words we used were naturally spoken by a male native Mandarin speaker from northern China and were recorded using Praat (Boersma & Weenink, 2020) with a 44.1 kHz sampling rate and 16‐bit resolution. Four continua were generated using the TANDEM‐STRAIGHT MATLAB toolbox (Kawahara & Morise, 2011). The TANDEM‐STRAIGHT extracted the F0 structure, aperiodicity, and spectrogram information of two endpoint stimuli (e.g., /i/ with Tone 1 and Tone 2 in the tone continuum) as the sources and filter parameters. Using the source‐filter model and the specified number of steps, TANDEM‐STRAIGHT morphed a continuum from source A (corresponding to one endpoint) to source B (corresponding to the other endpoint) in the temporal domain for the tone and aspiration continua, and in the frequency and temporal domains for the vowel and transition continua (Kawahara et al., 2009). Each continuum included nine stimuli and each stimulus had a duration of 350 ms. Figure 1 shows the schematic diagrams of the four continua.

Stimuli in the tone continuum differed in F0. The syllables /i/ with Tone 1 (stimulus 1, “衣”) and /i/ with Tone 2 (stimulus 9, “姨”) were selected as the two endpoints. The F0 onset of stimulus 1 was 151 Hz, and that of stimulus 9 was 109 Hz, as shown in Figure 1a. The onset of F0 decreased from 151 to 109 Hz at a step size of approximately 5 Hz.

Stimuli in the vowel continuum differed in the first and second formants (F1, F2). Consonant‐vowel structure syllables /tu/ (stimulus 1, “嘟”) and /ti/ (stimulus 9, “滴”) were chosen as endpoints. The F1 frequency of stimulus 1 was 384 Hz, and the F2 frequency of stimulus 1 was 1002 Hz. The F1 frequency of stimulus 9 was 282 Hz, and the F2 frequency of stimulus 9 was 2273 Hz. As presented in Figure 1b, the F1 frequency changed from 384 to 282 Hz, with a step size of 13 Hz. The F2 increased from 1002 to 2273 Hz, with a step size of 159 Hz.

The VOT varied across the stimuli in the aspiration continuum. Syllables /pa/ (stimulus 1, “八”) and /p^h^a/ (stimulus 9, “趴”) were selected as the two endpoints. As shown in Figure 1c, the VOT of stimulus 1 was 6 ms, and that of stimulus 9 was 76 ms. The step size of VOT between two adjacent stimuli was approximately 8.75 ms.

Transition frequencies varied for stimuli in the transition continuum. Syllables /pa/ (stimulus 1, “八”) and /ta/ (stimulus 9, “搭”) were chosen as the two endpoints. The duration of the transition was 40 ms. The F2 onset frequency of stimulus 1 was 1207 Hz, and that of stimulus 9 was 1554 Hz, as presented in Figure 1d. The step size of F2 onset between two adjacent stimuli was approximately 43.38 Hz.

### Procedure

We first conducted the experiment on the 6‐ and 10‐year‐old children. Based on their results, we then asked all the children and adolescents to complete the tone perception task; only children aged 4–10 years were involved in the vowel perception task; and only children aged 6–10 years and adolescents aged 14 years were involved in the aspiration and transition perception tasks (see more details in Supporting Information). All the adults finished the perceptual tasks for the four continua. The order of the continua was counterbalanced across the participants. All the participants were required to complete the identification and discrimination tasks in a quiet room using a laptop with E‐Prime 2.0 installed. The sound stimuli were presented to participants via an earphone. Before the experiment, the intensity of each stimulus was normalized to 70 dB SPL in Praat, and the volume was adjusted to a comfortable level for each participant.

In the identification task, participants were required to quickly assess whether the sound stimulus was Sound 1 (e.g., “衣” in the tone continuum) or Sound 2 (e.g., “姨”), and to press the corresponding keys: Key “1” for Sound 1 and key “2” for Sound 2. The 4‐year‐old children were illiterate and hence unable to press the keys by themselves. Researchers first taught them to label Sound 1 (e.g., a picture of clothes for “衣” in the tone continuum on the left of a computer screen) and Sound 2 (e.g., a picture of a middle‐aged woman for “姨” on the right of a computer screen). The researchers then helped them to press the corresponding key after they had pointed at a picture to indicate their choice. There were nine repetitions of each stimulus in the testing block for adults, whereas to avoid fatigue there were only five repetitions for the children and adolescents. There were 324 trials for the adults (i.e., 4 continua × 9 stimuli × 9 repetitions) and 180 trials for the children and adolescents (i.e., 4 continua × 9 stimuli × 5 repetitions) in total. All the stimuli were presented randomly.

In the discrimination task, participants were asked to quickly assess whether two sequentially presented stimuli were the same (pressing key “1”) or different (pressing key “2”; AX pattern). Researchers asked the 4‐year‐old children to make their judgments and helped them to press the corresponding keys. The interstimulus interval was 500 ms. A total of 23 pairs of stimuli were included: 9 stimuli paired with themselves (i.e., 1‐1, 2‐2, 3‐3, … 8‐8, 9‐9) and 14 pairs of different stimuli separated by two steps in forward order (i.e., 1‐3, 2‐4, 3‐5, … 7‐9) and reverse order (i.e., 3‐1, 4‐2, 5‐3, … 9‐7). All pairs of stimuli were presented randomly and repeated five times for all participants. There were 460 trials in total (i.e., 4 continua × 23 pairs × 5 repetitions).

To help familiarize the participants with the identification and discrimination tasks, practice blocks with feedback were carried out before the testing blocks. The practice block for the identification task used only stimulus 1 and stimulus 9. To ensure that participants had understood the tasks, the testing blocks were released only when they obtained an accuracy above 90% in the practice blocks.

### Data analysis

For the identification task, we analyzed responses for Sound 1 in the four continua. We conducted a Probit analysis on the identification responses to calculate the position of the category boundary and the boundary width (Finney, 1971). We defined the boundary position as the 50% crossover point of the identification curve, and the boundary width was the linear distance between the 25th and 75th percentiles of the identification curve. A narrower boundary meant a sharper transition between phonological categories and thus reflected a clearer distinction of phonological categories.

For the discrimination task, all the pairs of stimuli were grouped into seven comparison units, each of which included four types of stimuli pairs: A‐A, B‐B, A‐B, and B‐A (e.g., 1‐1, 3‐3, 1‐3, and 3‐1). The discrimination accuracies of seven comparison units were calculated using the formula proposed by Xu et al. (2006): *P* = *P* (‘*S*’|*S*) × *P* (*S*) + *P* (‘*D*’|*D*) × *P* (*D*). In this formula, *P* (*S*) is the percentage of the same stimuli pairs (e.g., 1‐1, 3‐3) and *P* (*D*) refers to the percentage of different stimuli pairs (e.g., 1‐3, 3‐1). Here, *P* (‘*S*’|*S*) and *P* (‘*D*’|*D*) respectively index the percentage of “the same” responses to the same stimuli pairs, and the percentage of “different” responses to different stimuli pairs. On the basis of the boundary position, discrimination accuracies were further divided into between‐category accuracy and within‐category accuracy. The between‐category accuracy was the average accuracy of two comparison units straddling the categorical boundary. The within‐category accuracy was the average accuracy of the remaining comparison units. Discrimination peakedness indicates one's ability to enhance between‐category discrimination and inhibit within‐category discrimination (Xu et al., 2006), and here it referred to the difference between within‐category accuracy and between‐category accuracy.

By considering the potential individual differences across participants (Barr et al., 2013), linear mixed‐effect models (LMMs) were created to assess the identification performance and discrimination performance of all groups in R (R Core Team, 2019), using the lme4 package (Bates et al., 2015). Bonferroni correction was adopted for multiple comparisons.

## RESULTS

### Identification and discrimination of vowels /u/−/i/

**FIGURE 2:**
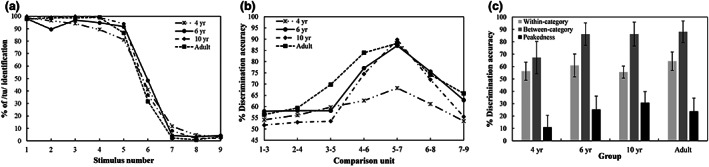
(a) Identification curves, (b) discrimination curves, (c) discrimination accuracy and peakedness of vowels in the children and young adults. Error bars = ±1 *SD*.

Figure 2a shows the identification responses for vowel /u/, in the children and young adults. A steep change in the response score was observed from /u/ to /i/ in the identification curve for each group. We created an LMM with boundary width as a dependent variable, and the model consisted of a fixed factor of *group* and a random factor of *subject* [boundary width ~ group + (1|subject)]. The results demonstrated that the effect of *group* was not significant (*F* = 2.475, *p* = .0679, *η* ^2^ = .09; 4‐year‐olds: *M* = 1.799; 6‐year‐olds: *M* = 1.801; 10‐year‐olds: *M* = 1.004; adults: *M* = 1.313). An LMM was also conducted with boundary position as a dependent variable, *group* as a fixed factor, and *subject* as a random factor [boundary position ~ group + (1|subject)]. The results revealed that the effect of *group* was not significant (*F* = 0.7464, *p =* .5278, *η* ^2^ = .03; 4‐year‐olds: *M* = 5.766; 6‐year‐olds: *M* = 5.783; 10‐year‐olds: *M* = 5.922; adults: *M* = 5.683). These findings indicate that the performance of vowel identification was similar for the four groups.

Figure 2b shows the discrimination curves for vowels: An accuracy peak was found around the category boundary of vowels in each group. A linear mixed‐effect model was created with discrimination accuracy as a dependent variable. The model included two fixed factors of *group* and *category*, and a random factor of *subject* [discrimination accuracy ~ group * category + (1|subject)]. We found a significant effect of *category* (*χ* ^2^ = 169.99, *p* < .001, *η* ^2^ = .84) and of *group* (*χ* ^2^ = 35.81, *p* < .001, *η* ^2^ = .36), and a significant interaction effect (*χ* ^2^ = 31.64, *p* < .001, *η* ^2^ = .33). There was a significant difference between within‐category accuracy and between‐category accuracy in each group (all *p*s < .0001). There was also a significant difference between the 4‐year‐olds' group and the other three groups in between‐category accuracy (all *p*s < .0001), but the differences among the other three groups did not reach a significant level (all *p*s = 1.000). We also observed a significant difference in within‐category accuracy between the 4‐year‐old children and the adults (*β* = −.0806, *SE* = .0279, *p* = .0271), and between the 10‐year‐old children and the adults (*β* = −.0879, *SE* = .0267, *p* = .0074).

An LMM was calculated with discrimination peakedness as a dependent variable, and the model contained a fixed factor of *group* and a random factor of *subject* [discrimination peakedness ~ group + (1|subject)]. We observed a significant effect of *group* (*F* = 12.29, *p* < .0001, *η* ^2^ = .33). There was a significantly smaller peakedness in the children aged 4 years than that in the other three groups (all *p*s < .001); the differences among the other three groups were not significant (all *p*s > .1; see Figure 2c).

To summarize, the children aged 4 years could categorically identify Mandarin vowels /u/ and /i/ as well as the adults could, but their discrimination ability was immature. The 6‐year‐old children had acquired an adult‐level ability to discriminate the vowels /u/ and /i/ in a categorical manner.

### Identification and discrimination of Tone 1‐2

**FIGURE 3:**
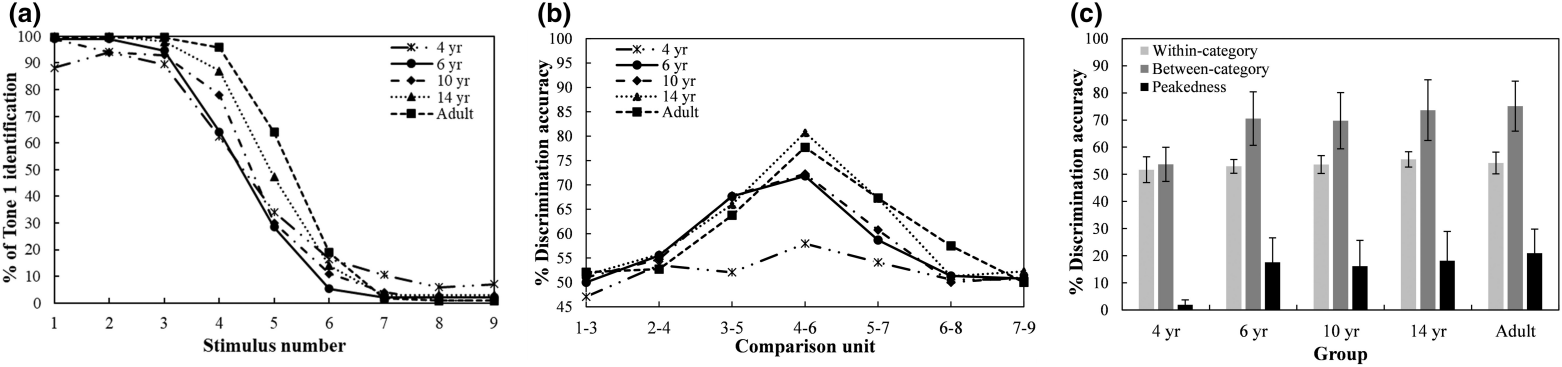
(a) Identification curves, (b) discrimination curves, (c) discrimination accuracy and peakedness of tones in children, adolescents, and young adults. Error bars = ±1 *SD*.

Figure 3a shows the identification responses to Tone 1 by the children, adolescents, and young adults. There was a sharp decrease in the identification response from Tone 1 to Tone 2 in each group. To assess the group difference in boundary width, we created an LMM with boundary width as a dependent variable. The model included *group* as a fixed factor and *subject* as a random factor [boundary width ~ group + (1|subject)]. There was a significant effect of *group* (*F* = 10.72, *p* < .0001, *η* ^2^ = .31), indicating that the children aged four (*M* = 2.689) had a significantly wider categorical boundary for tones than the other four groups did (6‐year‐olds: *M* = 1.322; 10‐year‐olds: *M* = 1.366; 14‐year‐olds: *M* = 1.329; and adults: *M* = 1.020; all *p*s ≤ .0001); the differences among the other four groups were not significant (all *p*s = 1.000).

For boundary position, an LMM [boundary position ~ group + (1|subject)] with *group* as a fixed factor and *subject* as a random factor (*F* = 8.975, *p* < .0001, *η* ^2^ = .27) again revealed a significant effect of *group*. The boundary position in the children aged 4 to 10 (4‐year‐olds: *M* = 4.561; 6‐year‐olds: *M* = 4.490; 10‐year‐olds: *M* = 4.608) was significantly closer to Tone 1 than that in the adults (*M* = 5.314; all *p*s ≤ .001). The difference in boundary position between the adolescents (*M* = 5.087) and the adults (*β* = −.2272, *SE* = .170, *p* = 1.000) and that between the 10‐year‐old children and the adolescents (*β* = −.4787, *SE* = .178, *p* = .0839) did not reach a significant level.

Discrimination curves for each tone comparison unit, and the between‐category accuracies and within‐category accuracies for the five groups, are presented in Figure 3b,c, respectively. A peak in discrimination accuracy occurred around the categorical boundary of tones in the 6‐ to 10‐year‐old children, the 14‐year‐old adolescents, and the adults. However, the between‐ and within‐category accuracies in the 4‐year‐old children were close to the chance level (50%). An LMM was created with discrimination accuracy as a dependent variable. The model contained two fixed factors—*group* and *category* (within‐category vs. between‐category), and a random factor—*subject* [discrimination accuracy ~ group * category + (1|subject)]. A significant effect of *group* (*χ* ^2^ = 45.52, *p* < .001, *η* ^2^ = .37) and *category* (*χ* ^2^ = 153.61, *p* < .001, *η* ^2^ = .74), as well as a significant interaction effect (*χ* ^2^ = 41.56, *p* < .001, *η* ^2^ = .34), were found. The difference between within‐category accuracy and between‐category accuracy in the children aged four was not significant (*β* = .0197, *SE* = .0221, *p* = .3737), but there was a significant difference between the within‐category and between‐category accuracies in the other four groups (all *p*s < .0001). The differences in within‐category accuracies across all groups were not significant (all *p*s = 1.000). The between‐category accuracy of the 4‐year‐old children was significantly lower than those of the other four groups were (all *p*s < .0001), whereas the differences across the other four groups were not significant (all *p*s > .1).

An LMM was also created with the peakedness of discrimination as a dependent variable; the model included a fixed factor of *group* and a random factor of *subject* [discrimination peakedness ~ group + (1|subject)]. The results indicated a significant effect of *group* (*F* = 12.24, *p* < .0001, *η* ^2^ = .34). The children aged 4 years had a significantly smaller peakedness than the other four groups did (all *p*s ≤ .0001), but the differences among the other four groups were not significant (all *p*s > .1).

To summarize, the 4‐year‐old children showed a poorer ability to identify and discriminate Mandarin Tone 1 and Tone 2 than the other four groups did. Furthermore, those aged six had acquired a relatively mature CP of Tone 1 and Tone 2.

### Identification and discrimination of consonants

### Consonant aspiration contrast /p/−/p^h^/

**FIGURE 4:**
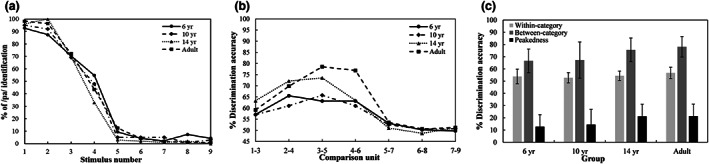
(a) Identification curves, (b) discrimination curves, (c) discrimination accuracy and peakedness of consonant aspiration in children, adolescents, and young adults. Error bars = ±1 *SD*.

Figure 4a presents identification responses for the unaspirated consonant /p/ in the four groups. Each group showed a sharp decrease in response scores from unaspirated consonant /p/ to aspirated consonant /p^h^/. We created an LMM with boundary width as a dependent variable, and the model contained a fixed factor of *group* and a random factor of *subject* [boundary width ~ group + (1|subject)]. The results indicated a significant effect of *group* (*F* = 3.885, *p* = .0121, *η* ^2^ = .13). The 6‐year‐old children (*M* = 1.995) had a significantly wider boundary width than the 14‐year‐old adolescents (*M* = 1.081; *β* = .914, *SE* = .298, *p* = .0180) and the adults did (*M* = 1.241; *β* = .753, *SE* = .286, *p* = .0608). However, the differences among the 10‐year‐olds' group (*M* = 1.643), 14‐year‐olds' group, and the adults' group (all *p*s > .3) were not significant. Furthermore, an LMM was calculated with boundary position as a dependent variable, and the model included a fixed factor of *group* and a random factor of *subject* [boundary position ~ group + (1|subject)]. The results revealed no significant effect of *group* (*F* = 0.3435, *p* = .7939, *η* ^2^ = .01; 6‐year‐olds: *M* = 3.813; 10‐year‐olds: *M* = 3.710; 14‐year‐olds: *M* = 3.571; adults: *M* = 3.808).

Discrimination curves for consonant aspiration are displayed in Figure 4b. In each group, we found a peak of discrimination accuracy around the category boundary predicted from the identification curve. The within‐ and between‐category accuracies, and the discrimination peakedness of the children, adolescents, and young adults, are shown in Figure 4c. An LMM was calculated with discrimination accuracy as a dependent variable, and the model contained two fixed factors of *category* and *group* and a random factor of *subject* [discrimination accuracy ~ group * category + (1|subject)]. The results revealed a significant effect of *group* (*χ* ^2^ = 19.17, *p* < .001, *η* ^2^ = .21) and of *category* (*χ* ^2^ = 129.93, *p* < .001, *η* ^2^ = .74), and also a significant interaction effect (*χ* ^2^ = 11.06, *p* = .011, *η* ^2^ = .13). In each group there was a significant difference between within‐category accuracy and between‐category accuracy (all *p*s ≤ .0001). Furthermore, the children aged 6 to 10 years had significantly lower between‐category discrimination accuracy than the adolescents and the adults did (both *p*s < .02), whereas the difference between the adolescents and the adults was not significant (*β* = −.0252, *SE* = .0253, *p* = 1.000).

An LMM was conducted with discrimination peakedness as a dependent variable, and the model contained a fixed factor of *group* and a random factor of *subject* [discrimination peakedness ~ group + (1|subject)]. The results indicated a significant effect of *group* (*F* = 3.752, *p* = .0142, *η* ^2^ = .13): the 6‐year‐olds' group had a marginally smaller peakedness than the adults' group did (*β* = −.0863, *SE* = .0323, *p* = .0553), but the differences among the other three groups were not significant (all *p*s > .1).

In summary, the CP of consonant aspiration (/p/−/p^h^/) in the 6‐year‐old children was immature. The 10‐year‐old children could maturely identify consonants /p/ and /p^h^/ in a categorical manner, but their discrimination ability was immature. The 14‐year‐old adolescents had acquired an adult‐level CP ability to identify and discriminate unaspirated consonant /p/ from aspirated consonant /p^h^/.

### Consonant formant transition contrast /p/−/t/

**FIGURE 5:**
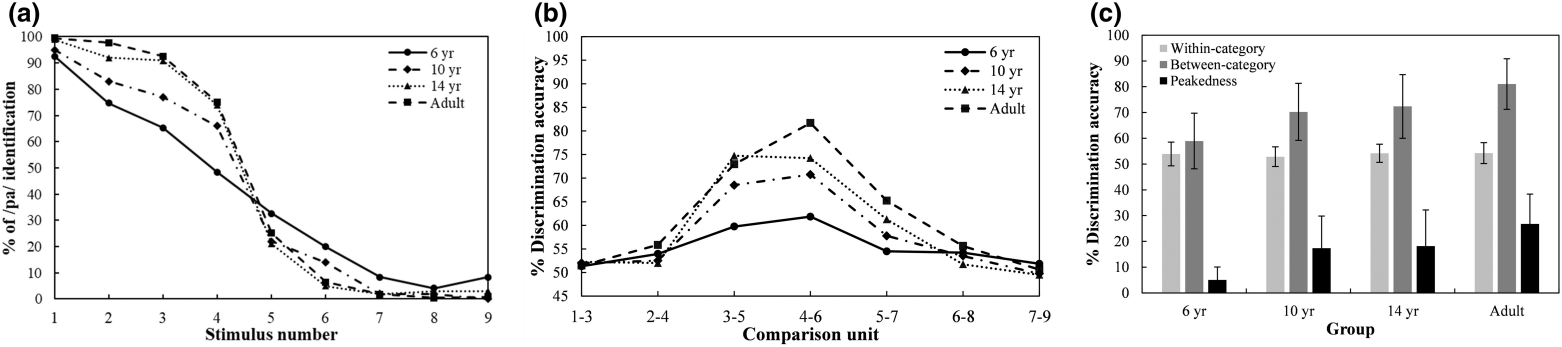
(a) Identification curves, (b) discrimination curves, (c) discrimination accuracy, and peakedness of consonant formant transitions in children, adolescents, and young adults. Error bars = ±1 *SD*.

Figure 5a shows the identification responses for consonant /p/ in the four groups. A steep decrease in the response score from consonant /p/ to /t/ could be observed in the children aged 10, the adolescents aged 14, and the adults. However, there was a continuous and gradual change of response score in the children aged six. An LMM was conducted with boundary position as a dependent variable. The model consisted of a fixed factor of *group* and a random factor of *subject* [boundary position ~ group + (1|subject)]. The results revealed no significant effect of *group* (*F* = 1.229, *p* = .3048, *η* ^2^ = .05; 6‐year‐olds: *M* = 3.942; 10‐year‐olds: *M* = 4.076; 14‐year‐olds: *M* = 4.393; adults: *M* = 4.512). The LMM conducted for boundary width [boundary width ~ group + (1|subject)] did indicate a significant effect of *group* (*F* = 14.73, *p* < .0001, *η* ^2^ = .36). The 6‐year‐olds' group (*M* = 2.801) had a significantly wider categorical boundary than the other three groups did (10‐year‐olds: *M* = 1.753; 14‐year‐olds: *M* = 1.459; adults: *M* = .968; all *p*s < .004). There was also a significant difference in boundary width between the children aged 10 and the adults (*β* = .785, *SE* = .277, *p* = .0353), but the differences were not significant between the 10‐year‐old children and the adolescents (*β* = .294, *SE* = .290, *p* = 1.000), or between the adolescents and the adults (*β* = .491, *SE* = .277, *p* = .4842).

The between‐category discrimination accuracy and the within‐category discrimination accuracy of consonant formant transitions in each group is displayed in Figure 5c. We conducted an LMM with discrimination accuracy as a dependent variable, and the model contained two fixed factors of *category* and *group* and a random factor of *subject* [discrimination accuracy ~ group * category + (1|subject)]. There was a significant effect of *group* (*χ* ^2^ = 35.99, *p* < .001, *η* ^2^ = .36) and of *category* (*χ* ^2^ = 121.71, *p* < .001, *η* ^2^ = .67), and also a significant interaction effect (*χ* ^2^ = 34.68, *p* < .001, *η* ^2^ = .30). The difference was not significant between the within‐category accuracy and the between‐category accuracy in the 6‐year‐old children (*β* = .0505, *SE* = .0268, *p* = .0631), whereas in the other three groups, within‐category accuracy was significantly lower than between‐category accuracy was (all *p*s < .0001). Furthermore, the differences in within‐category accuracy across the four groups were not significant (all *p*s = 1.000), but the children aged 6 to 10 and the adolescents showed significantly lower between‐category accuracy than the adults did (all *p*s ≤ .005).

An LMM was conducted with discrimination peakedness as a dependent variable. The model consisted of a fixed factor of *group* and a random factor of *subject* [discrimination peakedness ~ group + (1|subject)]. The results indicated a significant effect of *group* (*F* = 11.25, *p* < .0001, *η* ^2^ = .30): there was a significant difference between the 6‐year‐olds' group and the other three groups (all *p*s < .015). However, the differences among the other three groups were not significant (all *p*s > .08).

In summary, the 6‐ to 10‐year‐old children could not perceive consonants /p/ and /t/ maturely in a categorical way. However, the adolescents aged 14 years had acquired an adult‐level CP ability to identify and discriminate between the different consonant formant transitions for /p/ and /t/.

## DISCUSSION

**FIGURE 6:**
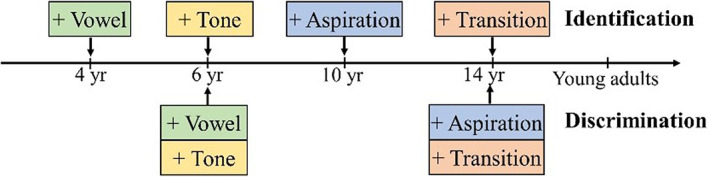
Maturation of identification and discrimination for different types of Mandarin phonemic contrasts. A “+” means that the ability has already become mature.

This is the first study to systematically uncover the maturation of CP in children and adolescents in regard to Mandarin segments and suprasegments (i.e., lexical tone contrast Tone 1‐2, vowel contrast /u/−/i/, consonant aspiration contrast /p/−/p^h^/, and consonant formant transition contrast /p/−/t/) with different temporal or spectral features, and different static or dynamic acoustic features. Of particular note is that we tested identification and discrimination abilities simultaneously. We found that the 4‐year‐old children could identify Mandarin vowel contrast /u/−/i/ as well as the adults could in a categorical manner, whereas their discrimination of vowel contrast /u/−/i/ and CP of lexical tone contrast Tone 1‐2 were immature. The 6‐year‐old children, however, had obtained an adult‐level CP ability to identify and discriminate vowel contrast /u/−/i/ and lexical tone contrast Tone 1‐2, whereas their CP of consonants (/p/−/p^h^/ and /p/−/t/) was immature. The 10‐year‐old children had mature CP of vowel contrast /u/−/i/ and lexical tone contrast Tone 1‐2, and they could identify consonant aspiration contrast /p/−/p^h^/ as well as the adults could in a categorical manner, whereas their discrimination of consonant aspiration (/p/−/p^h^/) and their CP of consonant formant transitions (/p/−/t/) were immature. The 14‐year‐old adolescents had acquired an adult‐level CP ability to identify and discriminate all the contrasts used in this study. Figure 6 shows the timeline for the maturation of identification and discrimination abilities of Mandarin vowel contrast /u/−/i/, lexical tone contrast Tone 1‐2, consonant aspiration contrast /p/−/p^h^/, and consonant formant transition contrast /p/−/t/. We will discuss the development of each contrast in detail next.

For the CP of vowel contrast /u/−/i/, the 4‐year‐old children in our study showed a mature ability of identification, as was demonstrated by the fact that the age differences we detected in boundary width and boundary position were not significant. This timepoint is earlier than that found by Arai et al. (2008), who showed a significant improvement in vowel identification among 6‐year‐old children. A possible reason for the inconsistent findings is that the acoustic variation of vowels in Arai et al.'s (2008) study (/a/−/a:/) was duration, whereas in our study (/u/−/i/) it was formant frequency, thus suggesting that the perceptual development of temporal and spectral cues may be different. In addition, we found that the 4‐year‐old children differed from adults in their ability of vowel discrimination, and that the discrimination of vowel contrast /u/−/i/ became mature in the children aged six—a timepoint that is consistent with that reported in Lee et al. (2012). This finding also suggests, in agreement with the findings of Chen et al. (2017) that phonemic identification and discrimination abilities follow different developmental courses. Thus, our findings support the need to assess these two abilities simultaneously when investigating CP.

For the CP of lexical tone contrast Tone 1‐2, native listeners above the age of 6 years showed a steep change in identification responses, a peak of discrimination accuracy around the category boundary, and a significant difference between their within‐ and between‐category accuracies. These findings indicate that the children older than 6 years could perceive Mandarin Tone 1 and Tone 2 categorically, according to the characteristics of CP set out by Repp (1984). However, the difference between within‐category accuracy and between‐category accuracy was not significant in the 4‐year‐old children, demonstrating that they could not perceive Tone 1 and Tone 2 categorically. In accordance with the findings of Chen et al. (2017), our study found that the 6‐year‐old children could perceive Mandarin tone contrast Tone 1‐2 as well as the adults could. However, we observed an age difference in the boundary position, which had not been reported in previous studies. Indeed, we found a trend showing that the boundary position moved from Tone 1 to Tone 2 with age. In the children aged below 10, the boundary position was significantly closer to Tone 1 than it was in the adolescents and the adults. Wang (1976) proposed two kinds of boundary: a psychophysical boundary for listeners who lack tonal language experience, and a linguistic boundary for native tonal language listeners. In Wang's study, phonological knowledge enabled listeners with the linguistic boundary to distinguish between the level tone and rising tone, whereas those with the psychophysical boundary could only differentiate level tone and non‐level tone according to acoustic signal differences; their boundary position was therefore closer to the level tone. This finding suggests that inadequate knowledge, and experience of the native language, might account for the difference in boundary position in children younger than 10. Our results further suggest that the maturation of CP depends on the participants' accumulative native language experience.

For the CP of consonant aspiration contrast /p/−/p^h^/, each group showed a steep change in identification responses, a peak of discrimination accuracy around the category boundary, and a significant difference between within‐category accuracy and between‐category accuracy. These results demonstrate that all the participants could perceive consonant aspiration contrast /p/−/p^h^/ categorically. Nonetheless, identification ability in the children aged six was poorer than that of the adults, whereas the children aged 10 had acquired a mature identification ability for consonant aspiration contrast /p/−/p^h^/ which was equal to that of the adults. The immature identification ability of consonant aspiration in the 6‐year‐olds and the mature identification ability of the 10‐year‐olds in our study are consistent with the findings of Xi et al. (2009), who observed immature identification of consonant aspiration in 7‐year‐old Mandarin‐speaking children. However, our findings were inconsistent with those of Hazan and Barrett (2000), who reported that the identification of initial consonants with differing VOT was immature in English‐speaking children aged 12. A possible explanation might be that the differences in VOT for voiced and voiceless English consonants (e.g., 50 ms in Hazan & Barrett, 2000) are much smaller than the VOT differences for aspirated and unaspirated Mandarin consonants (e.g., 70 ms in this study). This language difference may account for the relatively early maturation in the identification of aspirated and unaspirated consonants in Mandarin compared with that for voiced and voiceless consonants in English. Moreover, Hazan and Barrett (2000) and Xi et al. (2009) tested identification ability only, and did not test discrimination ability. We found that discrimination ability in the children aged 10 was poorer than that of the adults—a finding that was consistent with those of Ma et al. (2021). We also found that the adolescents aged 14 years had reached an adult level in their ability to discriminate the aspiration of Mandarin consonants. Ours is the first study to report the timepoint for the maturation of consonant aspiration discrimination in Mandarin.

For the CP of consonant formant transition contrast /p/−/t/, our findings of a lack of a steep change in the identification curve and a lack of a significant difference between within‐ and between‐category accuracies in the 6‐year‐old children indicated that they could not categorically perceive the consonant formant transitions in Mandarin, whereas listeners aged above 10 could perceive the transition continuum categorically. However, a significantly larger boundary width and lower between‐category accuracy in the children aged 10 revealed that they had not obtained a CP ability equal to that of the adults—a finding that is consistent with that of Hazan and Barrett (2000), who found that children aged 12 could not perceive the consonant formant transitions as well as adults could. We further found that the adolescents aged 14 years had achieved an adult‐level ability to identify and discriminate the consonant formant transition contrast /p/−/t/. Ours is the first study to report the timepoint for the maturation of consonant formant transition perception.

Our study observed an improvement with age in the CP abilities of children and adolescents. Nittrouer and Miller (1997) proposed a model of developmental weighting shift that assumed different weights were assigned to several properties of acoustic signals to perceive phonemes efficiently. Their model also assumed that children could develop the weighting scheme with accumulated language experience by shifting the weights assigned to different properties and ultimately could maturely perceive phonemes as well as adults can. Thus, accumulated language experience would enable children to focus on the critical features of acoustic signals in different phonetic environments and to gradually establish an adult‐level perceptual weighting scheme (Nittrouer & Miller, 1997). Those authors also believed that children's improved understanding of the relation between phonetic environment and phonetic informativeness guided the development of weighting scheme. Phonetic informativeness refers to the importance of certain properties in conveying phonetic information in the native language. Another possible explanation could be that explicit instruction on the metalinguistic knowledge of Mandarin phonemes in primary school and middle school can account for such improvement. In China, children in kindergarten receive no formal explicit teaching on phonemic knowledge, and that might lead to the poor performance of phonemic identification and discrimination in 4‐year‐old children.

Although language experience and phonemic knowledge certainly play an important role in the development of CP, we also consider it necessary to examine perceptual development within a broader cognitive science framework, with the thought that such findings might be helpful in explaining the developmental cognitive mechanism underlying speech perception. CP recruits working memory, in which the phonological store is an essential component of the phonological loop (Baddeley, 2007). The phonological store is used to maintain speech information temporarily, and its capacity and processing speed increase with age in children, so that older children can memorize more speech information and do so with higher efficiency than younger children can (Henry, 2012). Faster processing speed can also help children to quickly access long‐term phonological representations in categorical speech perception (Henry, 2012).

Our study also suggests that the development of the ability for identification is asynchronous with that for discrimination. We know that phonemic identification and discrimination require different cognitive resources (Fujisaki & Kawashima, 1971; Xu et al., 2006). For phonemic identification, listeners need to decode one sound stimulus and map the sound to long‐term developed phonemic knowledge. For phonemic discrimination (e.g., the AX pattern in this study), listeners need to memorize the first sound until the release of the second sound and then compare the two sounds. Thus, phonemic discrimination places a higher demand on working memory than identification does. The process of phonemic discrimination is more complex and requires greater cognitive resources, and those factors might explain why the children in our study had more difficulty acquiring discrimination ability than identification ability. Another possible explanation could relate to the different durations of the identification and discrimination tasks. For example, in the experimental design for children, the identification task for each continuum lasted approximately 6 min, whereas the discrimination task lasted 15 min. Although categorical speech perception can be automatic in adults (Zheng et al., 2014), it is a controlled process in children and requires their focused attention (Gomes, Molholm, Christodoulou, et al., 2000; Gomes, Molholm, Ritter, et al., 2000). Previous research has delineated a progressive development of sustained attention in children by the age of 10, with their sustained attention becoming mature after 10 years of age (Betts et al., 2006; Levy, 1980). Therefore, the fact that the maturation of discrimination ability was slower than that of identification might be correlated with the children's poor sustained and focused attention for long‐duration tasks.

Furthermore, we found that the perceptual ability for different types of phonemic contrasts had different maturation phases. Those findings are consistent with previous observations (e.g., Lee et al., 2012) that the perceptual development of tones and vowels with higher saliency was earlier and easier than consonant perception, supporting the phonological saliency hypothesis (Zhu & Dodd, 2000). In addition, this developmental order could be attributed to the feature of acoustic signals. Compared with tones and vowels (i.e., 350 ms in this study), the transitions (i.e., 40 ms) and aspiration (i.e., 6–76 ms) of consonants changed rapidly. Such a rapid change might increase the perceptual difficulty in children whose auditory systems are not mature. Although the human auditory system is reported to be precocious (Kisilevsky et al., 2009)—for example, fetuses can recognize their mother's voice during the prenatal stage—the maturation of the auditory system indeed follows a long and slow developmental course after birth. Postnatal development of the auditory system lasts a decade because of the immaturity of the middle ear and the gradual development of auditory transmission through the brainstem (D. R. Moore, 2002a; J. K. Moore, 2002b; J. K. Moore & Linthicum, 2007; Litovsky, 2015; Werner, 2007). Several studies also have attempted to determine the impact of general auditory sensitivity development on speech perception in children (Holt & Lotto, 2008; Nittrouer & Crowther, 1998; Wong & Cheng, 2020). For example, some brain‐imaging studies have found that musical training can strengthen general sound processing in children and improve their perception of tones, vowels, and consonants (Nan et al., 2018; Wong et al., 2007). Further research would help to unify the development of speech perception and general auditory or cognitive processing in an effort to uncover their underlying mechanisms.

### Limitations

This study uncovered the CP development, in children and adolescents, of Mandarin lexical tone contrast Tone 1‐2, vowel contrast /u/−/i/, consonant aspiration contrast /p/−/p^h^/, and consonant formant transition contrast /p/−/t/. However, it is oversimplified to generalize the findings of a few phonological contrasts to the entire phonological system. For example, the distinction of some phonological contrasts (e.g., Tone 2‐Tone 3) is more difficult than that of others (e.g., Tone 2‐Tone 4), so their developmental courses may be different. Similarly, we only tested the contrast between a front vowel and back vowel without a low vowel. More phonological contrasts should be taken into consideration in further research. Furthermore, considering the differences between child voice and adult voice, children may rely on different features for CP. Future studies should consider the role of talker variation in the CP development of children.

## CONCLUSION

In the current study, we found a long and slow maturation process of CP with accumulated language experience in Mandarin‐speaking children and adolescents. Children aged 4 years could already identify Mandarin vowels /u/ and /i/ as well as adults could in a categorical manner. Children aged 6 years had achieved an adult‐level ability to categorically identify and discriminate tones (Tone 1 and Tone 2) and vowels (/u/ and /i/). Children aged 10 years were able to identify the unaspirated stop /p/ and the aspirated stop /p^h^/ maturely in a categorical way. Adolescents aged 14 could categorically perceive stops differing in aspiration (/p/ and /p^h^/) and consonant formant transitions (/p/ and /t/) as well as adults could. Our observations that the CP of tones and vowels were earlier and easier to achieve support the phonological saliency hypothesis, which assumes that the lower saliency of consonants in the Mandarin phonological system leads to their later acquisition. The children's abilities in identification and discrimination showed different developmental trajectories. Thus, it will be necessary for further explorations of the development of CP to examine the two abilities simultaneously.

## FUNDING INFORMATION

This research was partly supported by the National Natural Science Foundation of China (11974374) and a fellowship award from the Research Grants Council of the Hong Kong SAR, China (Project No. PolyU/RFS2122‐5H01).

## Supporting information


Appendix S1.
