# Supplementary material for: Development of categorical speech perception in Mandarin‐speaking children and adolescents
Source: Child Dev. 2022 Aug 3;94(1):28–43. doi: 10.1111/cdev.13837 (PMC10087708; doi:10.1111/cdev.13837)
Supplement: Supplementary file 1 — Appendix S1. [file CDEV-94-28-s001.docx]

Table S1 lists the features of Mandarin lexical tone contrast Tone 1-2, vowel contrast /u/-/i/, consonant aspiration contrast /p/-/p^h^/, and consonant formant transition contrast /p/-/t/.

**TABLE S1** Features of Mandarin lexical tone contrast Tone 1-2, vowel contrast /u/-/i/, consonant aspiration contrast /p/-/p^h^/, and consonant formant transition contrast /p/-/t/.

| **Phonemic contrasts** | **Segment/**  **suprasegment** | **Spectral/**  **temporal cue** | **Static/**  **dynamic cue** | **High/low frequency** | **Rapid/slow change** |
| --- | --- | --- | --- | --- | --- |
| Lexical tone contrast Tone 1-2 | Suprasegment | Spectral | Static for level tone; Dynamic for contour tone | Low | Slow |
| Vowel contrast /u/-/i/ | Segment | Spectral | Static | High | Slow |
| Consonant aspiration contrast /p/-/p^h^/ | Segment | Temporal | Dynamic | N/A | Rapid |
| Consonant formant transition contrast /p/-/t/ | Segment | Spectral | Dynamic | High | Rapid |

Note: N/A is ‘not applicable’.

Here we explained why some tasks were not conducted for 4-year-old children and 14-year-old adolescents:

We firstly conducted the experiment on 6- and 10-year-old children and found that children with the ages of 6 and 10 had already categorically perceived Mandarin vowel contrast /u/-/i/ as adults, so we speculated that the results of the CP of this vowel contrast in the 14-year-old adolescents may not add more new information to our conclusion. Therefore, we did not conduct the task for the CP of vowels /u/-/i/ in the 14-year-old adolescents. Similarly, the results of 6- and 10-year-old children indicated that their CP of consonant contrasts /p/-/p^h^/ and /p/-/t/ was immature, so we speculated that the CP of these consonant contrasts in the 4-year-old children may be worse, which may not help us to find the maturation timepoint of the CP of consonants. Therefore, we did not conduct the tasks for the CP of the consonant contrasts in the 4-year-old children. In this case, we could shorten the duration of the whole experiment in some groups and avoid them being too tired during the experiment. The results of 6- and 10-year-old children are shown in Figure S1, which have not been reported/published before.


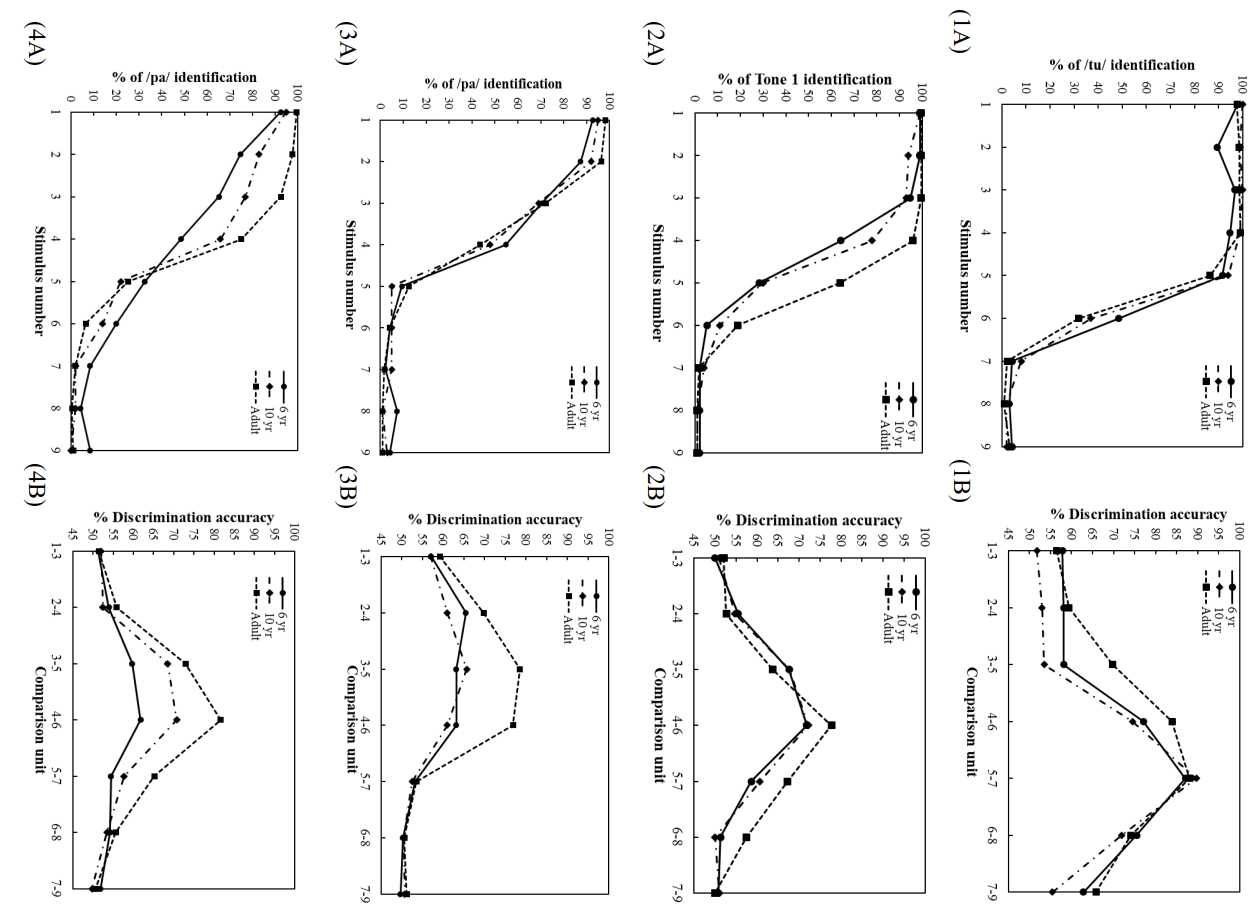


**FIGURE S1.** (A) Identification curves, and (B) discrimination curves of (1) vowel continuum, (2) lexical tone continuum, (3) consonant aspiration continuum, and (4) consonant formant transition continuum in six- and ten-year-old children and young adults.
